# Comparing generative artificial intelligence platforms and nursing student performance on a women’s health nursing examination in Korea: a Rasch model approach

**DOI:** 10.3352/jeehp.2025.22.23

**Published:** 2025-09-05

**Authors:** Eun Jeong Ko, Tae Kyung Lee, Geum Hee Jeong

**Affiliations:** 1 School of Nursing and Research Institute in Nursing Science, Hallym University, Chuncheon, Korea; 2 Kangwon National University Hospital, Chuncheon, Korea; The Catholic University of Korea, Korea

**Keywords:** Artificial intelligence, Nursing education, Nursing students, Psychometrics, Republic of Korea

## Abstract

**Purpose:**

This psychometric study aimed to compare the ability parameter estimates of generative artificial intelligence (AI) platforms with those of nursing students on a 50-item women’s health nursing examination at Hallym University, Korea, using the Rasch model. It also sought to estimate item difficulty parameters and evaluate AI performance across varying difficulty levels.

**Methods:**

The exam, consisting of 39 multiple-choice items and 11 true/false items, was administered to 111 fourth-year nursing students in June 2023. In December 2024, 6 generative AI platforms (GPT-4o, ChatGPT free version, Claude.ai, Clova X, Mistral.ai, Google Gemini) completed the same items. The responses were analyzed using the Rasch model to estimate the ability and difficulty parameters. Unidimensionality was verified by the Dimensionality Evaluation to Enumerate Contributing Traits (DETECT), and analyses were conducted using the R packages irtQ and TAM.

**Results:**

The items satisfied unidimensionality (DETECT=–0.16). Item difficulty parameter estimates ranged from –3.87 to 1.96 logits (mean=–0.61), with a mean difficulty index of 0.79. Examinees’ ability parameter estimates ranged from –0.71 to 3.15 logits (mean=1.17). GPT-4o, ChatGPT free version, and Claude.ai outperformed the median student ability (1.09 logits), scoring 2.68, 2.34, and 2.34, respectively, while Clova X, Mistral.ai, and Google Gemini exhibited lower scores (0.20, –0.12, 0.80). The test information curve peaked below θ=0, indicating suitability for examinees with low to average ability.

**Conclusion:**

Advanced generative AI platforms approximated the performance of high-performing students, but outcomes varied. The Rasch model effectively evaluated AI competency, supporting its potential utility for future AI performance assessments in nursing education.

## Graphical abstract

[Fig f6-jeehp-22-23]

## Introduction

### Background

Since the public release of ChatGPT, a conversational generative artificial intelligence (AI) platform, on November 30, 2022 [1], public fascination with large language models has surged. In response to OpenAI’s ChatGPT, numerous international information technology companies have launched their own generative AI platforms, including Claude.ai by Anthropic [2], Clova X by Naver [3], Mistral.ai by Mistral AI [4], and Gemini by Google [5]. Research on these diverse generative AI platforms has proliferated rapidly.

In the biomedical field, efforts to evaluate the performance of these tools have intensified. The literature search using generative AI as a keyword reveals 2 primary research domains. The first assesses the accuracy of generative AI in responding to queries, evaluating its utility in licensure examinations, academic assessments, and the provision of medical information. For example, generative AI has achieved passing scores on the United States Medical Licensing Examination [6]. At Hallym University in Korea, one study compared generative AI performance with that of medical students on a parasitology exam, reporting a score of 60 out of 100 [7]. The second domain explores the use of generative AI in academic writing, including topic selection, paraphrasing, summarization, translation, English proofreading, peer review, automated drafting, and manuscript editing [8]. While earlier commercial AI programs were domain-specific, today’s generative AI platforms achieve similar performance on nursing-related tasks with only slight variations in accuracy and response style.

Although generative AI has reached a significant level of proficiency in examinations, it is not yet flawless. Moreover, prior studies evaluating generative AI performance on examinations have been predominantly based on classical test theory (CTT), focusing on total scores [6]. No studies have yet compared generative AI ability parameters using item response theory (IRT). To our knowledge, no previous research has incorporated item‐level response data to examine AI performance, creating a gap in the literature. Leveraging IRT-based analyses to estimate generative AI ability could improve performance evaluations for a representative range of platforms, offering a robust framework for future research.

### Objectives

This study aimed to compare the ability parameters of multiple generative AI platforms with those of nursing students at Hallym University on a women’s health nursing examination. Additionally, this study estimated item parameters to assess whether the performance of generative AI platforms varies across different item difficulty levels. The specific objectives were as follows: first, to determine whether the response data satisfy the unidimensionality assumption required for applying IRT; second, to estimate and compare the ability parameter estimates of students and generative AI using IRT-based item analysis; and third, to derive the test characteristic curve (TCC), test information curve (TIC), and depict the ability parameter positions of generative AI on a Wright map.

## Methods

### Ethics statement

Since the data are fully anonymized and contain no personal identifiers such as name, student ID, or other unique codes, the research can be exempt from institutional review board under the Bioethics and Safety Act (https://elaw.klri.re.kr/eng_mobile/viewer.do?hseq=33442&type=part&key=36).

### Study design

This psychometric study compared the ability and item parameter estimates derived from the Rasch model, an IRT framework, for a nursing examination.

### Setting

The examination comprised 50 items from a women’s health nursing course at Hallym University’s School of Nursing: 39 one-best-option multiple-choice questions and 11 true/false questions. The examination items and the correct answers were documented in Supplement 1. This final examination was administered to fourth-year nursing students in June 2023. On December 4, 2024, the identical items were presented to 6 generative AI platforms via prompts. The exact prompts to 6 generative AI platforms were as follows: For the following test items, write the most appropriate answer for each item on a single line in numerical order (items 1–39). For the next section, read each statement and indicate “1” if it is correct and “0” if it is incorrect (items 40–50). The AI responses were coded as binary data (1 for correct, 0 for incorrect). The AI platforms included GPT-4o (gpt-4o-2024-11-20) [1], ChatGPT free version (GPT-4o mini; 2024-07-18) [1], Claude 3 Sonnet [2], Clova X [3], Mistral.ai (Mistral large) [4], and Google Gemini (Gemini 1.5 Pro) [5].

### Variables

The primary outcome variables were the ability parameter estimates of the students and generative AI, together with the item parameter estimates, including the difficulty parameter estimates.

### Data sources

The women’s health nursing examination was administered to fourth-year nursing students at Hallym University in June 2023. The test included 39 one-best-option multiple-choice questions, 11 true/false items, and 15 constructed response items; only the 50 selected-response items were used for the generative AI evaluation. The answers to the constructed response items could not be used since the scores were not binary. Although the partial credit model could have been applied, it was considered overly complex for this study. Therefore, those 15 items were excluded from the analysis.

The examinee pool comprised 111 students and 6 generative AI platforms, totaling 117 examinees. Student and AI responses were combined into binary data to estimate item and ability parameters (Dataset 1). Items were categorized by content and detailed in Dataset 2.

### Measurement

Analysis was performed using the Rasch model. The unidimensionality assumption for IRT was verified using the Dimensionality Evaluation to Enumerate Contributing Traits (DETECT) method [9]. DETECT analysis was conducted on the item domain information. The domains of the items used for the analysis were derived from the “category” information in Dataset 2. The R ver. 4.5.0 (The R Foundation for Statistical Computing) code for this analysis is provided in Supplement 2. Estimation of examinee ability parameters and item parameters was performed using the R package irtQ (R ver. 4.5.1; 2025-06-13 ucrt) [10] with R code in Supplement 3. The Wright map was generated using the TAM R package [11], with R code provided in Supplement 4.

### Bias

No selection bias occurred, as all participant responses were analyzed.

### Study size

Sample size estimation was not performed, as all target students were included.

### Statistical methods

The results were described with descriptive statistics. The correlation analysis was performed using DBSTAT ver. 5 (http://dbstat.com/) [12].

## Results

### Unidimensionality assumption

The item domains were sourced from the “category” information in Dataset 2. DETECT results are presented in Table 1. The DETECT results were interpreted as shown in Tables 2 and 3 [13], confirming that the 50 nursing items exhibited essential unidimensionality.

MADCOV100, the mean absolute conditional covariance between score levels, indicates the overall strength of covariance between items. A value close to 1 (0.96) supports unidimensionality. MCOV100, the mean conditional covariance including signs, is expected to be negative under a unidimensional model. The observed negative value (–0.21) further supported unidimensionality.

### Item parameter estimation

**Fig. 1.**
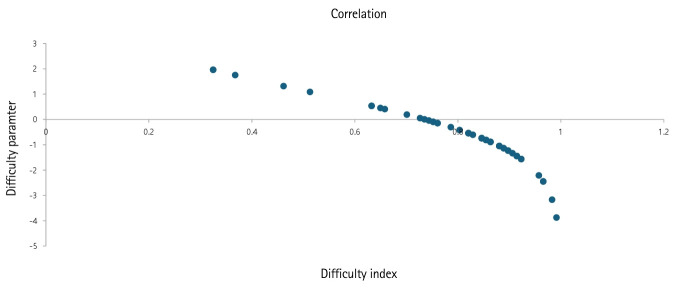
Correlation between the difficulty parameter estimates and difficulty index of the 50 items of a women’s health nursing examination at Hallym University, June 2023 (111 nursing students) and December 2024 (6 AI).

Difficulty parameter estimates for the 50 items were provided in Supplement 5. The estimates ranged from –3.87 to 1.96 logits, with a mean of –0.61 and a median of –0.73. In CTT, the difficulty index (correct proportion) ranged from 0.32 to 0.99, with a mean of 0.79 and a median of 0.85 (Supplement 5). The correlation between the estimates of the difficulty parameters and difficulty index was calculated as r=0.8991 (95% confidence interval [CI], 0.8577 – 0.9594; P<0.0001) (Fig. 1).

### Ability parameter estimation

**Fig. 2.**
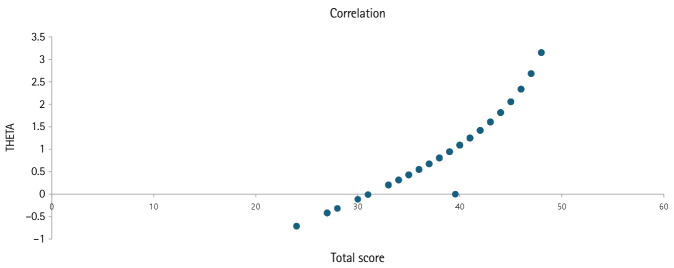
Correlation between the estimated theta value (THETA) and the total score (TOTAL_SCOR) of a women’s health nursing examination at Hallym University, June 2023 (111 nursing students) and December 2024 (6 AIs).

The estimates of the ability parameters of the examinees were detailed in Supplement 6. Estimates of the ability parameters ranged from –0.71 to 3.15 logits, with a mean of 1.17 and a median of 1.09. In CTT, the total scores ranged from 24 to 48 (maximum 50), with a mean of 39.6 and a median of 40 (Table 4). The Pearson correlation coefficient between the estimate of the ability parameter and total score was (r=0.9716; 95% CI, 0.9593–0.9802; P<0.0001) (Fig. 2). Two-parameter logistic models could not be performed due to the limited number of examinees.

### Item characteristic curve

Intraclass correlation coefficients for all items can be generated by modifying the item numbers in the R code in Supplement 3.

### Item information curve

IICs for all items can be generated by modifying the R code in Supplement 3.

### Test characteristic curve

**Fig. 3.**
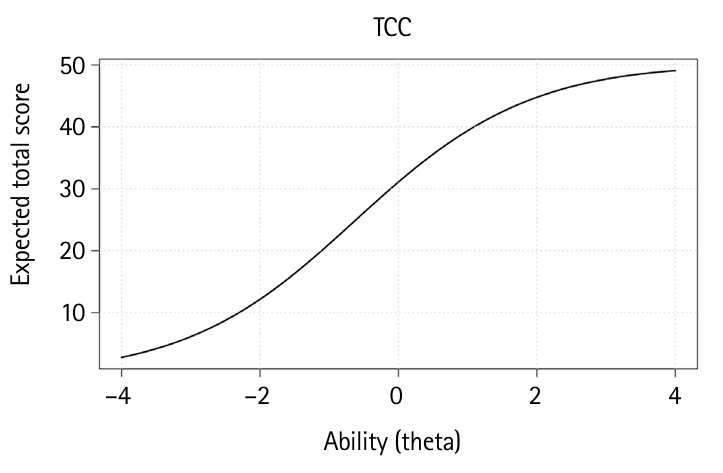
The test characteristic curve (TCC) for a women’s health nursing examination with 50 items by 117 examinees.

The TCC for this examination is depicted in Fig. 3.

### Test information curve

**Fig. 4.**
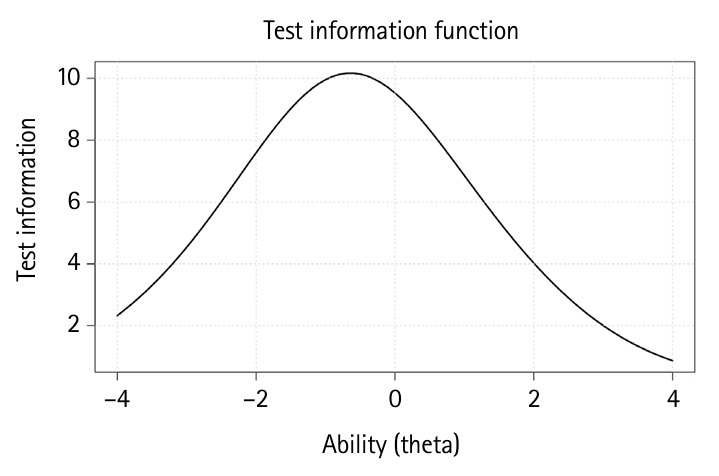
The test information curve for a women’s health nursing examination with 50 items by 117 examinees.

The test information curve for this examination is depicted in Fig. 4.

### Wright map (person ability item map)

**Fig. 5.**
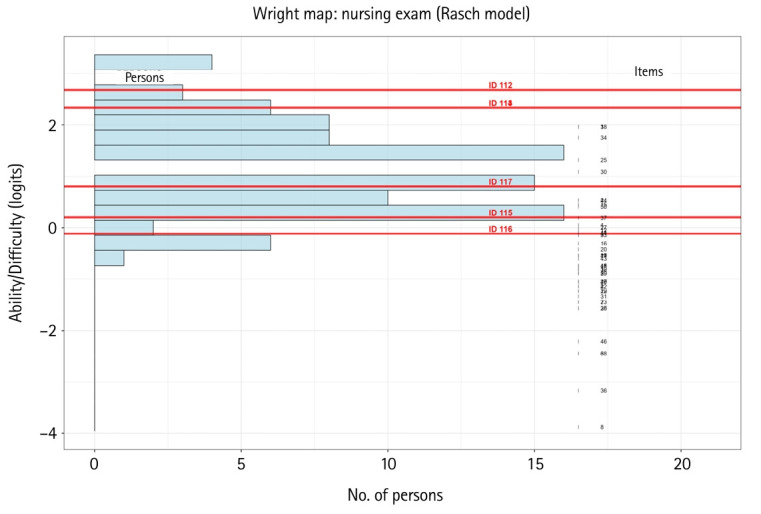
Wright map illustrating the ability parameters of each generative artificial intelligence platform. Red horizontal lines: examinees 112–117 at their THETA (ability) locations.

The Wright map, shown in Fig. 5, illustrates the ability parameter positions of each generative AI.

### Comparison of generative AI and nursing student ability parameters

As depicted in the Wright map (Fig. 5), 3 generative AI platforms (GPT-4o, ChatGPT free version, Claude.ai) exhibited ability parameter estimates above the median examinee value of 1.09, while 3 other platforms (Mistral.ai, Clova X, Google Gemini) were below it. This pattern aligns with the CTT total scores, where the first 3 exceeded the median proportion correct of 0.80, whereas the latter 3 fell below it.

## Discussion

### Key results

Among the 6 generative AI platforms evaluated, 3 demonstrated ability parameter estimates that exceeded the median examinee value, while 3 exhibited lower values. This dichotomy mirrors the comparisons based on the CTT correct proportion.

### Interpretation

In the 50-item women’s health nursing examination, the performance of generative AI platforms varied significantly. Some generative AI models exhibited ability parameters close to or below the median student ability, indicating limited knowledge or inconsistent problem-solving in this domain. These findings suggest that specific generative AI platforms have the ability to solve one-best multiple-choice questions and the true-and-false questions related to women’s health nursing. Additionally, the performance level of each generative AI model can be assessed according to IRT.

The ability of advanced generative AI to approximate the performance of high-performing students suggests its potential to calibrate item difficulty and inform targeted curriculum revisions. Additionally, integrating AI with IRT frameworks could enable adaptive testing platforms and individualized feedback systems, thus improving assessment precision and supporting personalized learning pathways in nursing education.

Furthermore, to propose the use of generative AI as virtual examinees to adjust item difficulty, it is necessary to approximate the ability spectrum of generative AI through multiple AI models or various attempts using different “prompt difficulty” levels. However, the variability observed in generative AI performance indicates that only the most advanced models are reliable at this level, whereas less proficient systems may struggle, limiting their value as standalone evaluators.

A large standard error (SE) in an examinee’s ability estimate, such as the SE of 0.62 for GPT-4o, reflects substantial uncertainty in the measured ability and reduces the reliability of individual-level interpretation. High SEs often arise when test items are not well targeted to the ability level or when response patterns are inconsistent. In AI models like GPT-4o, elevated SEs may reflect erratic guessing behaviors or response strategies misaligned with genuine proficiency. Such patterns can artificially inflate ability estimates, leading to an overestimation of competence. Therefore, ability estimates with large SEs should be interpreted cautiously, and additional measures—such as reviewing response patterns or applying stricter scoring criteria—should be considered to ensure valid inferences.

TCC shows the total expected test score according to the ability level. The curve increases monotonically, indicating that as examinee ability (θ) increases, the expected score also increases. The steepest part of the curve is below θ=0, meaning that the test is more sensitive (i.e., best at differentiating) for examinees with average ability. This pattern suggests that the test is well-targeted to the ability level of the low to average student. At the lower and upper ends of θ, the curve flattens, indicating that students with very low ability are likely to score near the minimum and those with very high ability will score near the maximum. The test has limited ability to discriminate among students at these extremes.

TIC indicates the precision of the test measurement according to each level of ability. The curve reaches its maximum (about 10) at below θ=0, indicating that the test is more informative and reliable for examinees with low to average ability. Information decreases symmetrically as θ moves away from 0 (with lower or higher ability). This means that the test provides less precise measurements for students with very low or very high abilities. The curve shape suggests that the exam is well-targeted for the low to low to average student population. It is less effective at reliably differentiating students at the extreme ends of the ability distribution.

 In Wright map (Fig. 5), the red bars highlight the ability estimates for particular examinees (labeled with examinee numbers). These selected examinees are spread across the ability continuum, from below –1 to above +1. The map enables a visual inspection of where these specific examinees fall relative to the overall ability distribution. For example, examinees 115 (Clova X), 116 (Mistral.ai), and 117 (Gemini) are below average (θ <–1); while examinees 112 (GTP4o), 113 (GPT free version), and 114 (Claude.ai) are above average (θ >1). This can help identify students who may need additional support or who are excelling. The map can be used to inform tailored feedback or interventions for specific students, especially those at the extremes. The overall spread of abilities covered by the histogram suggests that the exam is well matched to the range of abilities present in this cohort of examinees. There are no significant gaps in the ability distribution, indicating that the exam is capable of discriminating effectively among a wide range of students. This Wright map provides a clear visual summary of the ability distribution among 117 examinees, with selected individuals (highlighted by red bars) shown in the context of their peers. It enables educators to pinpoint where particular students are located on the ability scale and supports data-driven instructional decisions. The balanced spread of abilities also suggests that the exam is generally appropriate for the student population.

Due to the limited number of examinees, discrimination parameters could not be estimated, precluding an analysis of whether generative AI reliably answers highly discriminating items.

The examination items were presented in Korean. Nonetheless, Clova X, optimized for Korean language processing, achieved an ability parameter of 0.20, below the median, surpassing only Mistral.ai. This contrasts with previous reports claiming Clova X’s superior relevance (100%) in Korean queries compared to Google Bard (now Gemini) and Claude.ai [14]. This discrepancy suggests that the overall model architecture and training data coverage outweigh language-specific optimization.

### Comparison with previous studies

No comparable studies have evaluated the performance of generative AI or large language models using IRT-based models. In the nursing domain, a cross-sectional study of licensure-style questions reported that ChatGPT-4 achieved approximately 88% accuracy on an NCLEX-RN-type examination, significantly outperforming ChatGPT free version (79%) and Google Bard (64%) [15]. This superior performance of advanced GPT models aligns with our finding that at least one generative AI platform achieved near-expert proficiency. The utility of generative AI in educational assessment varies by exam content and model, with larger models increasingly approximating human examinee performance [16]. These previous findings support our results.

### Limitations

This study has several limitations. First, the human examinee sample was drawn from a single Korean university’s nursing program, which could limit generalizability. Variations in educational background and preparedness between institutions and regions can affect outcomes.

Second, the assessment was limited to a 50-item multiple-choice examination in women’s health nursing, excluding items of constructed response. Thus, the results apply only to selected-response knowledge questions and do not capture AI or student performance on open-ended explanations, clinical reasoning essays, or other critical assessment formats in nursing education.

Third, the 6 AI platforms evaluated represent a snapshot of current models, and their knowledge bases, often optimized for English, may not fully align with Korean-language nursing curricula except ClovaX. Although the examination was conducted in Korean, differences in language processing may have influenced the results, limiting generalizability to other areas or languages.

Finally, methodological constraints precluded the use of a 2-parameter logistic model so item discrimination and guessing parameters could not be estimated, and the precision of ability estimates is limited. The omission of a guessing parameter means that an AI lacking true knowledge might select correct answers by chance. While Rasch analysis facilitated relative ability comparisons, absolute interpretations of the “ability” of AI warrant caution.

### Suggestion for further research

The limitations of the study—a single cohort sample, a focused exam domain, the exclusion of open-ended assessments, and simplifying measurement assumptions—require future research to expand on this exploration of generative AI in nursing education evaluation. Future research should explore diverse content areas, incorporate open items, and employ advanced IRT models to improve AI competency assessments, fostering their integration into nursing education.

### Implications

These findings assist educators in determining the accuracy of generative AI when used for student instruction, and inform its integration into educational settings. A correlation was established between examinees’ scores under CTT and ability parameters under IRT, validating the use of both frameworks. The study also examined whether Korean-language specialized AI platforms offered advantages when tested in Korean, but no such advantage was found.

Score distributions and Rasch-derived abilities indicate that IRT, including the Rasch model, is viable to evaluate the performance of generative AI. Evaluating generative AI or large language models is labor-intensive, requiring extensive item development. However, implementing computerized adaptive testing based on IRT could streamline this process by efficiently estimating AI abilities, reducing time and item production efforts.

### Conclusion

This study demonstrated that advanced generative AI platforms, particularly GPT-4o, ChatGPT free version, and Claude.ai achieved ability parameters comparable to high-performing nursing students on a women’s health nursing examination, while others lagged. The Rasch model effectively estimated the ability and item parameters, revealing the suitability for low- to average ability examinees due to its skewed difficulty distribution. These findings indicate the potential of generative AI as a benchmark for calibrating item difficulty and supporting educational evaluation, although performance variability requires model-specific evaluations.

## Figures and Tables

**Figure f6-jeehp-22-23:**
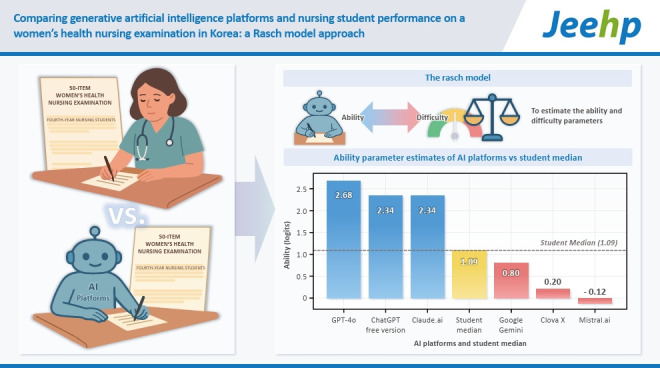


**Table 1.** Results of the unidimensionality test using DETECT

Variable	Unweighted	Weighted
DETECT	–0.160	–0.160
ASSI	–0.140	–0.140
RATIO	–0.166	–0.166
MADCOV100	0.961	0.961
MCOV100	–0.210	–0.210

DETECT, Dimensionality Evaluation to Enumerate Contributing Traits; ASSI, approximate simple structure index; RATIO, covariance ratio index; MADCOV100, mean absolute conditional covariance; MCOV100, mean conditional covariance.

**Table 2.** Interpretation of DETECT results

Interpretation	Level
Strong multidimensionality	DETECT >1.00
Moderate multidimensionality	0.40< DETECT <1.00
Weak multidimensionality	0.20< DETECT <0.40
Essential unidimensionality	DETECT <0.20

DETECT, Dimensionality Evaluation to Enumerate Contributing Traits.

**Table 3.** Interpretation of ASSI and RATIO for the unidimensionality test

Interpretation	Level	Level
Maximum value under a simple structure	ASSI=1	RATIO=1
Essential deviation from unidimensionality	ASSI>0.25	RATIO>0.36
Essential unidimensionality	ASSI<0.25	RATIO<0.36

ASSI, approximate simple structure index; RATIO, covariance ratio index.

**Table 4.** Ability parameters and total scores of 6 generative artificial intelligence platforms

Generative AI	Ability parameter	Error of the ability parameter	Total score (maximum score 50)	Proportion correct
GPT-4o	2.68	0.62	47	0.94
ChatGPT free version	2.34	0.55	46	0.92
Claude.ai	2.34	0.55	46	0.92
Clova X	0.20	0.33	33	0.66
Mistral.ai	-0.11	0.32	30	0.60
Google Gemini	0.80	0.36	38	0.76

## Footnotes

**Data availability**
Data files are available from https://doi.org/10.7910/DVN/PWV6H2 Dataset 1. Binary response data for 50 items from 117 examinees, including 111 nursing students and 6 generative AI platforms.

Dataset 2. Domains of items of the nursing examination.

## Supplementary materials

Supplement 1. 50 items of the women’s health nursing examination in Korean.

Supplement 2. The R code for DETECT to test unidimensionality.

Supplement 3. R code to estimate examinee ability parameters, item parameters, item characteristic curves, item information curves, test characteristic curves, and test information curves using the irtQ and TAM R packages.

Supplement 4. R code for the positions of the Wright map illustrating the ability parameters of each generative artificial intelligence (AI).

Supplement 5. Estimated item difficulty parameters of 50 items.

Supplement 6. Estimated ability parameters and total scores of 117 examinees. Generative AI’s ID were as follows: 112 GPT4o, 113 ChatGPT free version, 114 Claude.ai, 115 ClovaX, 116 Mistral.ai, and 117 Google Gemini.

Supplement 7. Audio recording of the abstract.
