# Supplementary material for: Comparing generative artificial intelligence platforms and nursing student performance on a women’s health nursing examination in Korea: a Rasch model approach
Source: J Educ Eval Health Prof. 2025 Sep 5;22:23. doi: 10.3352/jeehp.2025.22.23 (PMC12770907; doi:10.3352/jeehp.2025.22.23)
Supplement: Supplementary file 3 — Supplement 1. 50 items of the women’s health nursing examination in Korean. [file jeehp-22-23-suppl1.pdf]

2023학년도 4학년 1학기 여성건강간호학 학기말시험

학번 : ________________ 이름 : ______________

반드시, 시험지에 **학번과 이름 기록해서** 답지와 함께 제출해주세요.
● 시험 배점: 객관식 50문항 50점, = 총 50점 만점

■ 다음에 답은 OMR 카드에 적어주세요(1~39번, 각 1점씩).

1. 규칙적 월경을 하던 18세 여자가 3개월 동안 무월경일 때 우선적으로 시행할 검사는? 5
① 황체호르몬 검사 ② 난포자극호르몬 검사
③ 에스트로겐 부하 검사 ④ 프로게스테론 부하 검사
⑤ 융모생식샘자극호르몬 검사

2. 8개월 전에 초경을 시작한 14세 여자가 월경 시작 후 1~2일 동안 심한 월경통으로 일상생활이 힘들다고 한다. 검진결과에서 특이한 병리 소견이 없을 때 월경통의 주요 원인은?5
① 무배란월경주기 ② 티록신 과다 분비
③ 프로게스테론 과다 분비 ④ 옥시토신 과다 분비
⑤ 자궁내막의 프로스타글란딘 과다 생성

3. 여고생이 월경전증후군증상을 호소하며 보건실에 왔을 때 완화를 위한 적절한 교육은? 1
① “비타민 B 복합제를 복용하도록 한다.”
② “증상이 심하면 호르몬 요법이 필요할 수 있습니다.”
③ “복숭아와 수박 같은 수분이 많은 과일 섭취를 줄이세요.”
④ “월경이 끝나면 바로 증상이 없어지니 걱정하지 마세요.”
⑤ “병원을 방문해서 정확한 원인을 확인하는 것이 필요합니다.”

4. 기능(부전)성 자궁출혈의 가장 흔한 원인은? 3
① 임신관련 질환 ② 갑상선 기능장애
③ 무배란성 자궁출혈 ④ 황체화호르몬의 상승
⑤ 자궁의 기질적 병변

5. 53세 여성이 갱년기로 불규칙한 월경주기와 심한 열감과 홍조등 신체 변화들을 걱정할 때 간호사의 적절한 설명은? 4
① 에스트론 수치가 급상승하면서 월경 주기가 짧아질 수 있습니다.
② 이런 증상은 황체화호르몬 소실의 가속화로 나타납니다.
③ 배란이 안 되므로 피임 없이 성생활을 할 수 있습니다.
④ 난포자극호르몬의 혈중 농도가 높아진 상태입니다.
⑤ 고밀도지질단백질이 증가될 수 있습니다.

6. 최근 심한 열감과 불안 증상으로 52세 여성이 폐경기 호르몬 대체요법 상담을 받은 뒤 약물요법을 잘 이해한 것으로 보이는 표현은? 5

① 수면장애가 있으면 대체요법을 받을 수 없습니다.
② 대체요법은 골다공증이 있을 때 시작하는 것이 좋습니다.
③ 대체요법을 받으면 질 출혈이 사라질 것입니다.
④ 대체요법을 받으면 유방암을 예방합니다.
⑤ 대체요법을 받으면 안면홍조는 덜해질 것입니다.

7. 35세 여성이 질출혈로 초음파검사를 받은 결과에서 여러개의 자궁내막폴립이 확인되었다.
우선적 중재로 고려될 수 있는 것은? 2
① 원추절제술 ② 자궁소파술 ③ 전자궁절제술 ④ 자궁동맥색전술 ⑤ 메트로니다졸 투여

8. 자궁근종으로 복강경자궁절제술후 2일째 된 여자가 견갑통으로 복부팽만감을 호소하였다.
활력징후는 정상 범위이고 질 출혈은 없었다. 간호사 설명으로 옳은 것은? 5
① “가스배출을 위해 관장이 필요합니다.”
② “복강 출혈로 응급수술이 필요합니다.”
③ “복부 감염으로 항생제 투여가 필요합니다.”
④ “수액 과다 공급으로 수액량을 줄이면 좋아집니다.”
⑤ “수술 시 주입한 가스로 인한 것이니 많이 움직이세요.”

9. 30세 여성이 특별한 증상은 없이 건강검진에서 자궁근종(3x3 cm 크기)이 확인되었다.
우선적 중재로 고려될 수 있는 것은? 5
① 근종절제술
② 단순자궁절제술
③ 부분자궁절제술
④ GnRH 호르몬 요법
⑤ 6개월마다 정기검진

10. 자궁근종이 발생할 가능성이 높은 여성은? 4
① 조기 폐경된 40세 여성
② 5회 임신 4회 출산한 여성
③ 자궁내장치를 삽입한 여성
④ 에스트로겐 피임약을 장기 복용한 여성
⑤ 만성 골반내감염 치료를 받는 여성

11. 50세 강씨는 월경과 월경 사이에 성관계를 하면 무통성 점적출혈이 있다고 하였다.
세포진검사에서 CIS(carcinoma in situ) 결과를 받았는데 의미하는 것은? 2
① 정상세포 ② 상피내암 ③ 비정형증 ④ 중증 이형증 ⑤ 침윤성 경부암

12. 자궁경부상피내종양의 중증 이형증(CIN Ⅲ)에 관한 설명으로 옳은 것은? 4
① 조기진단이 불가능하다.
② 침윤성암으로 방사선치료가 필요하다.
③ 단순포진 바이러스가 주요 유발요인이다.
④ 침윤성암의 전구 질환으로 CIS(carcinoma in situ)를 포함한다.
⑤ 정상 편평상피세포가 경증 궤양 변화를 일으킨 것이다.

13. 산과력 T2-P2-A0-L2인 42세 여자의 자궁경부질세포진검사(Pap test)에서 Class Ⅰ 이었다.

예상되는 추후 관리는? 5

① 즉시 원추절제술 ② 한 달 후 쉴러검사
③ 배란 시 질확대경검사 ④ 월경이 끝난 후 자궁내막 생검
⑤ 정기적인 자궁경부질세포진검사

14. 자궁경부암 치료로 내부 방사선 조사를 받는 대상자 간호는? 3

① 변비 예방을 위해서 고섬유성 식이를 제공한다.
② 정서 지지를 위해서 가족과 친구의 방문을 격려한다.
③ 방광팽만 예방을 위해서 유치 도뇨관을 삽입한다.
④ 활동을 제한하기 위해서 수분 섭취를 최소한으로 줄인다.
⑤ 요통이 있을 경우 자주 체위를 바꾸어 준다.

15. 48세 여성이 자궁경부암 IIa기로 진단을 받았다. 이 여성에게 권고되는 치료 방법은? 5

① 원추절제술 ② 전기소작술 ③ 전자궁적출술 ④ 환상투열요법 ⑤ 근치자궁절제술

16. 폐경이 안 된 42세 여성이 자궁근종과 왼쪽 난소종양으로 전자궁적출술과 좌측난소난관절제술을 시행하였다. 수술 후 교육으로 적절한 것은? 답 2

① "월경의 변화는 없을 것입니다."
② "에스트론겐 분비는 지속됩니다."
③ "두 달에 한 번 씩 배란이 될 것입니다."
④ "열감이나 발한 증상이 나타날 수 있습니다."
⑤ "성기능이 감소하여 성관계가 힘들 수 있습니다."

17. 25세 여성이 한 달 전 산부인과 외래에서 우측 난소에 5cm 낭종을 진단받았다. 3시간 전 부터 갑자기 극심한 복부 통증으로 응급실로 왔다. 검사결과, 혈압 60/50mmHg, 맥박 110회/분, 체온 37℃, 호흡 24회/분, WBC 8000/μL, Hb 8.2g/dl, Hct 25%였다.
의심되는 건강문제는? 5

① 난관수종 ② 난관임신
③ 골반내 감염 ④ 난소낭종 염전
⑤ 난소낭종 파열

18. 10세 전후 어린 여아에게 잘 발생하는 난소종양은? 1

① 생식세포종양 ② Brenner 종양
③ Krukenberg 종양 ④ 세르톨리 레디그 종양
⑤ 난소섬유종

19. 25세 여자가 왼쪽 난소낭종으로 낭종절제술을 받았다. 낭종 안에는 모발과 치아 등이 있었다. 수술 후 교육으로 옳은 것은? 1

① 월경 변화는 없다. ② 조기 폐경으로 호르몬대체요법이 필요하다.
③ 배란이 되지 않는다. ④ 매달 흉부 X선 검사를 한다.
⑤ 황체호르몬 치료가 필요하다.

20. "**양성**" 난소종양에 대한 설명으로 옳은 것은? 1
① 점액성 낭선종은 크기가 크다.
② 성관계시 질 출혈이 주요 증상이다.
③ 시간이 경과하면 악성종양으로 바뀐다.
④ 양측성으로 빈발하고 고형의 특성이 있다.
⑤ 과립막세포종은 이십대 무월경과 남성화가 주요 증상이다.

21. 자궁내막증식증으로 진단받은 여자가 월경과다증(hypermenorrhea)이 있을 때 치료 목적으로 투여하는 호르몬은? 3
① 옥시토신 ② 프로락틴
③ 프로게스테론 ④ 프로스타글란딘
⑤ 갑상샘자극호르몬

22. 월경통과 성교통이 있는 여성이 진단검진한 결과, 난소에서 자궁내막조직이 발견되었다. 예상되는 건강문제는? 3
① 난포낭종 ② 자궁선근증
③ 자궁내막증 ④ 자궁내막증식증
⑤ 다낭난소증후군

23. 42세 여성이 임신반응 검사에서 음성인데 자궁이 커져 있고 월경과다증과 월경곤란증이 심해진다고 한다. 가장 의심이 되는 건강문제는? 5
① 자궁외임신 ② 포도상기태
③ 자궁경부암 ④ 자궁내막암
⑤ 자궁선근증

24. 30세 여자가 다량의 농성 질분비물이 있으며 골반과 하복부에 심한 통증이 있고 체온은 38℃이다. 항생제 치료를 받고 있을 때 간호중재는? 1
① 반좌위를 취하게한다. ② 케겔운동을 권장한다.
③ 수분섭취를 제한한다. ④ 유치도뇨관을 삽입한다.
⑤ 저잔류식사를 제공한다.

25. 골반내감염인 여성의 퇴원교육에서 잠재적으로 위급한 후유증으로 강조할 것은? 4
① 만성골반통증 ② 불임
③ 우울증 ④ 자궁외임신
⑤ 자궁경관암

26. 외음, 질, 직장에 꽃양배추(Cauliflower)모양 사마귀가 특징인 성매개성질환의 원인균은? 5
① 포도상구균 ② 연쇄상구균
③ 디프테로이드 ④ 스피로헤타균
⑤ 인유두종바이러스

27. 칸디다성 질염에 대한 설명으로 옳은 것은 ? 5
① 질에 발적과 딸기모양 반점이 있다.
② 메트로디나졸을 경구투약한다.
③ 무증상인 성 파트너도 함께 치료받아야 한다.
④ 임신 중 감염되면 신생아 안염을 유발할 수 있다.
⑤ 항생제 복용과 당뇨병 등 유산간균의 감소로 발생한다.

28. 트리코모나스 질염에 효과적인 치료제는?   답 5
① 니스타틴 (nystatin)   ② 독시사이클린 (doxycycline)
③ 테트라사이클린 (tetracycline)   ④ 에리트로마이신 (erythromycin)
⑤ 메트로니다졸 (metronidazole; flagyl)

29. 25세 여성이 많은 양의 질 분비물과 질 부위 자극과 가려움으로 병원을 방문하였다. 분비물에서 우유 빛깔의 생선비린 냄새가 날 때 관련된 질염은?   답 3
① Toxoplamosis   ② Candidiasis
③ Bacterial vaginitis   ④ Trichomonas vaginitis
⑤ Group B-streptococcus

30. 단순포진 감염인 임부 관련 설명으로 틀린 것은? 3
① 성교, 피부접촉과 입맞춤으로 감염된다.
② 산도감염 예방하기 위해 제왕절개로 분만한다.
③ 임신 중에 빈발하고 치료가 늦으면 태아기형을 초래한다.
④ 감염 초기 acyclovir(아사이클로비르) 치료는 재발을 줄인다.
⑤ 좌우 대칭성 수포가 형성되고 통증이 심하다.

31. 방광질누공으로 복원수술을 받은 여성의 수술 후 상처부위 치료를 위한 간호중재는? 3
① 저단백식사를 제공한다.   ② 정맥주입량을 제한한다.
③ 유치도뇨관을 유지한다.   ④ 변이 질로 배출되는지 확인한다.
⑤ 다리를 움직이지 못하게 확인한다.

32. G6-P5 임신력인 70세 여성이 3도 자궁탈출증 진단을 받았을 때 예상되는 수술은? 4
① 원추절제술   ② 루프환상투열절제술
③ 맥도날드교정술   ④ 질식자궁절제술
⑤ 근치자궁절제술

33. 다음은 3년간 임신이 안 된 부부 중 남편이 받은 정액검사 결과이다. 검사결과에 대한 해석으로 옳은 것은? 1

·정액량: 2.5ml	·정자 수: 60,000,000개/ml
·운동성 정자의 비율: 70%	·pH: 7.5

① 정상 소견　　② 정자부족증
③ 정액량 부족　　④ 운동성 정자의 비율이 낮음
⑤ 정액의 높은 산성도

34. 난임 여성의 자궁내막검사에 대한 설명으로 옳은 것은? 2
① 난포의 크기를 측정한다.　　② 황체기능 결함을 확인한다.
③ 배란 후 2~3일째에 검사한다.　　④ 복강경 검사와 병행해서 시행한다.
⑤ 검사하기 3일 전부터 성관계를 금지한다.

35. 자궁경부와 양쪽 난관의 문제가 있는 난임 여성에게 우선적으로 고려할 수 있는 치료는? 4
① 보조 부화술　　② 생식세포 난관 이식
③ 접합자 난관 이식　　④ 체외수정
⑤ 대리모 임신

36. 자궁경부암(HPV) 백신에 대한 옳은 설명은? 3
① 20세 이후에 백신 접종하는 것이 안전하다.
② 국가에서 만 13~14세 여성에게 1회 접종을 권고한다.
③ 성 경험 시작하기 전에 백신 접종을 하는 것이 효과가 높다.
④ 서바릭스가 가다실보다 HPV 예방 효과가 크다.
⑤ HPV 예방접종을 통해 많은 유형의 HPV(2, 4, 6, 11, 18, 21)를 예방할 수 있다.

37. 자궁경부질세포진검사(Pap test) 권고에 대한 설명으로 **틀린 것은? 4**
① 임신 여부와 상관없이 검사받는다.
② 자궁경부암 예방백신 접종 여부와 상관없이 검사받는다.
③ 만 20세부터 검사를 시작하는 것을 권고한다.
④ 성생활을 하지 않는다면 5년마다 1회 검사받는다.
⑤ 자궁경부암 조기 발견에 비용 효과면에서 이득이 크다.

38. 자궁난관조영술(hysterosalpingography)으로 확인할 수 있는 것은? 2
① 자궁내막증　　② 나팔관의 개방
③ 정자의 운동성　　④ 난소의 성숙난포
⑤ 난소의 배란 유무

39. 성폭력 피해자에게 발생할 수 있는 원치 않는 임신을 예방하기 위한 중재는? 3
① 즉시 질 세척을 시행한다.
② 24시간 이내에 질살정제를 투여한다.
③ 72시간 이내에 응급피임약을 투여한다.
④ 임신반응검사 결과가 나올 때까지 기다린다.
⑤ 2~3주 동안 코르티코스테로이드 호르몬을 투여한다.

■ **다음을 읽고 맞으면 “1”, 틀리면 “2”로 답하시오 (40~50번, 각 1점씩).**

40. 2022년 국내 합계출산율은 “0.78”이다. 1

41. 젊은 여성이 월경주기는 정상인데 월경기간과 월경량이 감소하면 희발월경이다. 2

42. 젊은 여성에게 가장 흔한 질염은 세균성 질염이다. 1

43. 클라미디아는 남성보다 여성에게 흔한 성병이다. 1

44. 매독 1기의 특징적 증상은 연성하감이다. => 경성하감이다 정답 2

45. 자궁근종은 대부분 점막하 근종이 많다. 2

46. 자궁경부세포검사에서 비정상일 경우 질확대경검사를 실시한다. 1

47. 자궁내막증(endometriosis)은 자궁내막이 비정상적으로 과도증식된 상태이다. 2

48. 골반통증을 호소하는 난임 여성은 자궁근종일 가능성이 크다. 2

49. 간질성세포종양이 난소암의 80% 이상으로 가장 빈도가 높다. 2

50. 자궁경관 냉동치료(cryotherapy)후 질분비물이 다량 있을 경우 감염 가능성이 높다. 2

검정색: 2019학년 국시문제
파랑색: 2019학년 새로 출제문제
초록색: 2021년 국시, 새로 출제문제
